# Peri-Implant Soft Tissue Conditioning by Means of Customized Healing Abutment: A Randomized Controlled Clinical Trial

**DOI:** 10.3390/ma12183041

**Published:** 2019-09-19

**Authors:** Mario Beretta, Pier Paolo Poli, Silvia Pieriboni, Sebastian Tansella, Mattia Manfredini, Marco Cicciù, Carlo Maiorana

**Affiliations:** 1 Department of oral surgery, Fondazione Policlinico Ca’ Granda, 20141 Milan, Italy; dr.marioberetta@gmail.com (M.B.); poliodontoiatria@gmail.com (P.P.P.); carlo.maiorana@unimi.it (C.M.); 2 School of oral surgery, Fondazione Policlinico Ca’ Granda, 20141 Milan, Italy; silvia.pieriboni@unimi.it (S.P.); mattia.manfredini@unimi.it (M.M.); 3 Department of Oral Rehabilitation, Italian Institute of Stomatology, 20141 Milan, Italy; sebastian.tansella@gmail.com; 4 BIOMORF Department, University of Messina, 98100 Messina, Italy

**Keywords:** CAD/CAM, dental implant, emergence profile, soft tissue, custom healing abutment

## Abstract

Introduction: An optimal aesthetic implant restoration is a combination of a visually pleasing prosthesis and adequate surrounding peri-implant soft tissue architecture. This study describes a novel workflow for one-step formation of the supra-implant emergence profile. Materials and Methods: Two randomized groups were selected. Ten control group participants received standard healing screws at the surgical stage. Ten individualized healing abutments were Computer aided Design/Computer aided Manufacturing (CAD/CAM)-fabricated out of polyether ether ketone (PEEK) restoration material in a fully digital workflow and seated at the surgical stage in the test group. The modified healing abutment shape was extracted from a virtual library. The standard triangulation language (STL) files of a premolar and a molar were obtained considering the coronal anatomy up to the cement-enamel junction (CEJ). After a healing period ranging from 1 to 3 months depending on the location of the surgical site, namely, mandible or maxilla, a digital impression was taken. The functional implant prosthodontics score (FIPS) and the numerical rating scale (NRS) of pain were recorded and compared. Results: The mean FIPS value for the test group was 9.1 ± 0.9 while the control group mean value was 7.1 ± 0.9. In the test group, pain assessment at crown placement presented a mean value of 0.5 ± 0.7. On the contrary, the control group showed a mean value of 5.5 ± 1.6. Conclusions: Patients in the test group showed higher FIPS values and lower NRS scores during the early phases compared to the control group.

## 1. Introduction

Nowadays, fulfilling the aesthetic expectations of patients is one of the most challenging tasks in implant dentistry [1]. Factors that influence the aesthetic outcome of an implant-supported rehabilitation include, amongst others, the position of the implant [2], the quantity and quality of hard and soft tissues, and their adaptation over time [3].

Since the aesthetic result plays a pivotal role in the success of implant rehabilitations, several indices have been developed in order to evaluate the aesthetics of a single restoration over time [4]. These evaluations are based on photographs and models and give credit to the peri-implant soft tissue frame, showing its importance to the final aesthetic outcome [4]. A method that is commonly used to evaluate both the functional and aesthetic results is the functional implant prosthodontics score (FIPS) [5].

The development of ideal supra-implant soft tissue architecture can be achieved by step-wise conditioning using a provisional crown [6]. Conditioning of the soft tissues requires multiple sessions in which the shape of the temporary restoration is modified in order to condition the peri-implant mucosa according to the desired morphology. This approach however is time-consuming, since multiple appointments are necessary to modify the provisional crown. Moreover, continuous removal of the temporary prosthesis may lead to biological trauma of peri-implant soft tissues, thus jeopardizing the final aesthetic result [7].

In the present study, a novel digital workflow consisting of one-step formation of the supra-implant emergence profile using individualized healing abutment was performed. In accordance with Joda et al., the rationale was to increase the aesthetic result and reduce patient discomfort [8]. Thus, the aim of the present study was to prospectively evaluate the peri-implant soft tissue response to customized healing abutments compared to standard healing caps.

## 2. Materials and Methods

### 2.1. Study Design

The present study was designed as a monocentric randomized controlled clinical trial performed in a private practice setting. All surgical and prosthetic procedures were performed by the same clinician.

To be included in the study, patients had to present with good general health (ASA I and ASA II according to the American Society of Anesthesiologists Classification), and with mandibular or maxillary single-tooth gaps distal to the canines to be restored with standard implants, with no need for additional bone and soft tissue augmentation procedures. Implants had to be positioned with adequate primary stability so that transmucosal healing can be accomplished.

The following exclusion criteria were adopted: age < 18 years at time of surgery; poor oral hygiene condition (full mouth plaque score > 25% and full mouth bleeding score > 25%); abuse of alcohol or drugs; smoking habits (> 10 cigarettes/day); presence of acute oral infections; remote or recent radiation therapy in the oro-maxillo-facial area; recent chemotherapy; intake of drugs affecting bone metabolism; pregnancy. Implants showing signs of peri-implant mucositis and peri-implantitis during the study period were excluded from the following analyses.

Prior to randomization, a total of 20 participants were enrolled in the study. The gender ratio was 40% males (n = 8)/60% females (n = 12). The mean age of all included patients was 60.75 ± 12.58. A total of 3 premolars and 4 molars were restored in the maxilla, while 2 premolars and 11 molars were treated in the mandibula. 

Patients were allocated to two different experimental groups by simple randomization procedures (computerized random numbers). Subject randomization was conducted using a computer spread-sheet. On the day of the surgery each patient was assigned to the experimental groups according to sealed envelopes containing the type of treatment. The test group received modified healing abutments, while the control group received standard healing abutments.

Following randomization, the test and control groups were each composed of 6 females and 4 males subjects. The distribution of teeth in test and control groups is presented in Table 1.

No drop out was reported after randomization procedures up until the final follow-up recall. 

During the first appointment, the medical and dental histories of each participant were recorded. At the same time, clinical and radiological examinations were conducted to design a patient-specific treatment plan. Clinically, full-mouth plaque and bleeding scores were recorded and the amount of keratinized gingiva, and the stability of the remaining teeth. Every radiological factor that might potentially influence the treatment plan were assessed. Panoramic and peri-apical radiographs were used as a first level exam to evaluate the availability of bone before implant insertion. If a second level exam was needed to assess any bone deficiency, cone-beam computed tomography scan was performed. Finally, each patient underwent full-arch digital impression using an intraoral scanner (CS 3600, Carestream Dental ©, Carestream Health, Inc., Rochester, NY, USA). 

A signed informed consent was obtained from all patients involved in this research before starting with the surgical procedures. There was no drop out during both the surgical and prosthetic procedure.

Contrary to public and private health centers (DM 18/3/1998 published in the Official Gazette, GU n. 122 of 28-05-1998), Italian law does not require ethical committee approval for clinical work performed in private dental offices, and therefore no ethical committee resolution was released [9].

### 2.2. Surgical Protocol

All surgical procedures were performed by the same surgeon on an outpatient basis. Antibiotic prophylaxis was administered to the patients, consisting of amoxicillin clavulanate 2 g 1 h before surgery to reduce the risk of infections. Local anesthesia was induced with mepivacaine 2% with epinephrine 1:100.000. 

A mucoperiosteal flap was raised following middle crestal incision in the maxilla, whereas in the mandible the incision was carried out taking care to divide keratinized gingiva into equal parts on the buccal and lingual sides. Implant bed preparation was performed and implants (iSy Implant System^®^, Camlog Biotechnologies, Basel, Switzerland)) were inserted according to the manufacturer’s instructions.

All patients were treated with a single-stage surgical technique. In the control group, standard cylindrical polyether ether ketone (PEEK) healing abutments with conical connection were applied, while modified gingival formers were positioned in the test group.

**Figure 1:**
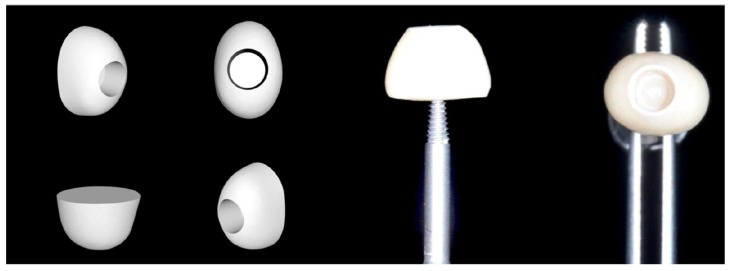
On the left: the elaboration of the STL file from a virtual library. On the right: the anatomically modified healing abutment.

The modified healing abutment shape was extracted from a virtual library (exocad^®^ DentalCAD, Fraunhofer IGD (Darmstadt, D)). The STL files of a premolar and a molar were obtained considering the coronal anatomy up to the cemento-enamel junction (CEJ). The STL files were then digitally cut to a height of 4 mm from the CEJ using the dental modelling software (Figure 1).

**Figure 2:**
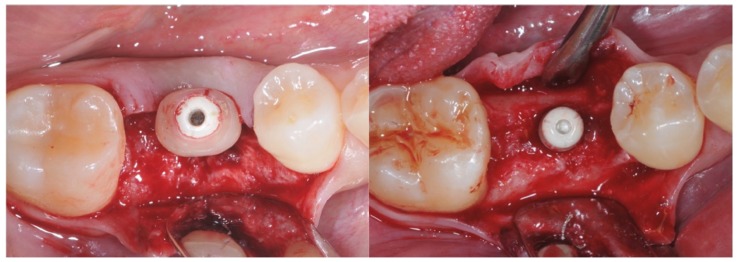
On the left: placement of the anatomically modified healing screw. On the right: placement of the standard healing screw.

At this point, it was possible to virtually align and combine the STL file of the internal part of the standard healing cap with the STL file of the sectioned tooth retrieved from the virtual library and cut 4 mm coronally to the cemento-enamel junction. The modified healing screw was finally produced with CAD/CAM milling from a PEEK block (BioHPP, Bredent^®^, Bolzano, Italy) (Figure 1) and screwed to the pre-mounted implant base thanks to the snap-on system in the test group (Figure 2). 

Conversely, standard gingival former screws were positioned in the control group (Figure 2). 

The flaps were then adapted to the healing abutments and sutured allowing transgingival healing with 4-0 sutures (CV-5, Gore-Tex^®^; W.L. Gore & Associates, Flagstaff, AZ, USA)), and an intraoral radiograph was taken.

### 2.3. Prosthetic Protocol

**Figure 3:**
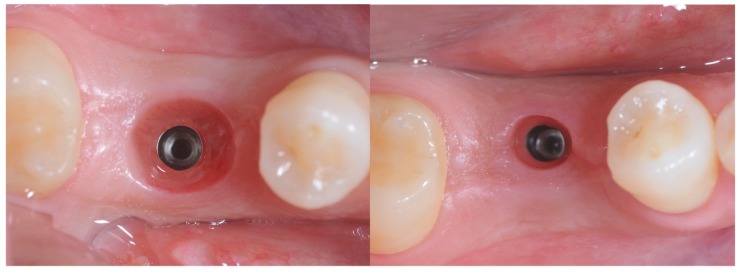
On the left: tissue conditioning achieved with the anatomically modified healing screw. On the right: tissue conditioning achieved with the standard healing screw.

Sutures were removed at the 12-day follow-up visit. After a healing period ranging from 1 to 3 months (Figure 3) depending on the location of the surgical site, namely, mandible or maxilla, respectively, a digital impression was taken using a multifunctional cap mounted on the implant base. 

**Figure 4:**
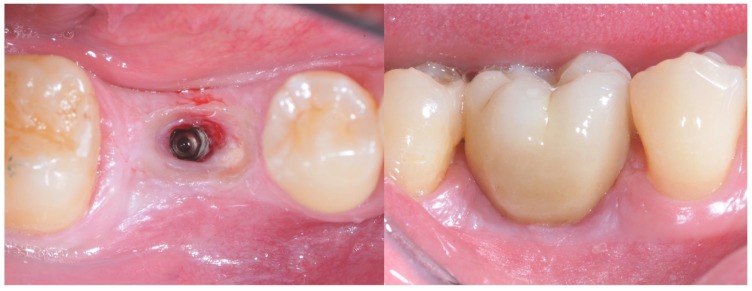
On the left: tissue distress at crown placement after conditioning with a standard healing screw. On the right: placement of the zirconia crown after tissue conditioning with a standard healing abutment.

**Figure 5:**
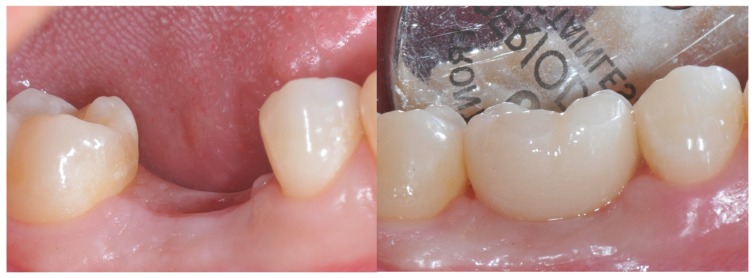
Placement of the zirconia crown after tissue conditioning with the anatomically modified healing abutment.

After one week, a screw-retained super-translucent multi-layer cubic monolithic zirconia crown (Katana^™^ Zirconia STML, Kuraray Noritake Dental Inc^©^, Okayama, Japan) was positioned (Figure 4 and Figure 5) and an intraoral radiograph was performed.

### 2.4. Study Outcomes

To clinically evaluate the soft tissues, the FIPS was used. The said score is defined by five variables: (1) interproximal, (2) occlusion, (3) design, (4) mucosa, and (5) bone. A scoring scheme of 0 – 1 – 2 was assigned for each aforementioned variable, resulting in a maximum score of ten (5 × 2) per implant reconstruction. 

After crown insertion, a questionnaire for the assessment of perceived pain was submitted to each patient. Each form contained the numerical rating scale (NRS), which consists of the personal evaluation of pain on a scale from 1 to 10 (one is the minimum pain and ten is the maximum pain) [10]. The questionnaire was proposed at three different study periods, namely at crown insertion (baseline), and after 2 and 24 h.

All FIPS measurements were collected by an independent examiner blinded to the type of evaluation in order to limit bias.

The same clinician that performed both surgical and prosthetic procedures, collected the NRS scores. The values were statistically evaluated using the non-parametric Wilcoxon sum rank test for unpaired samples.

## 3. Results

Overall, the results from 20 standard implants placed with a single-stage surgical technique in 20 patients were available for the statistical analysis. The test group received 10 anatomically modified gingival formers, while the control group received 10 standard healing abutments. The healing period was uneventful in all patients, and 20 multi-layered cubic monolithic zirconia crowns were delivered.

FIPS and NRS values were collected for both groups. The mean FIPS value for the test group was 9.1 ± 0.9, with a minimum score of 8.0 and a maximum score of 10.0. The control group mean value was 7.1 ± 0.9, with a minimum score of 6.0 and a maximum score of 8.0. The test group obtained higher FIPS scores than the control group. The difference was statistically significant (*p* = 0.0006).

According to the study design, pain assessment was recorded at different periods: at baseline (T0), and after 2 h (T1) and 24 h (T2).

At baseline, the test group presented a mean value of 0.5 ± 0.7, while, after 2 h and 24 h, the mean value was 0.

The control group showed a mean value of 5.5 ± 1.6 at crown placement. After 2 h and 24 h the mean values were 2.7 ± 1.6 and 0.4 ± 1.3, respectively. 

The difference between the two groups was statistically significant at the time of crown placement (*p* = 0.0001) and after 2 h (*p* = 0.0002), but not 24 h later (*p* = 0.3) (Table 2 and Table 3).

## 4. Discussion

A satisfying and predictable aesthetic result is a key element in modern implantology. Correct 3D implant positioning together with adequate quantity and quality of peri-implant hard and soft tissues are essential prerequisites to fulfill the aesthetic expectations of patients [3,11,12,13,14,15]. Moreover, the emergence profile plays a key role in the achievement of a satisfying aesthetic result [16].

Standard circular healing abutments, as stated by Janakievski in 2007, are unable to support peri-implant soft tissues and to create a natural profile [17]. The creation of the correct emergence profile often requires a gradual conditioning of tissues through progressively modified temporary restorations [18]. This stage consists in several sessions [19], which extend the treatment time and increase the final costs [20]. 

Repeated modifications of the provisional crown can also involve mechanical and biological trauma, damaging the epithelial attachment and leading to bacterial contamination [7].

The absence of a provisional step, in the case of thick biotypes, can lead to a difficult crown insertion. In such cases, excessive compression can cause ischemia and constitute a risk factor for future recession [7].

In order to overcome these drawbacks, several techniques have been proposed. According to Pow, a personalized healing abutment may be useful in creating an ideal soft tissue profile for definitive crown sitting [21]. In 2015, Raj et al. customized standard healing abutments by incrementally adding fluid composite [22]. This allowed the insertion of the definitive crowns without using temporary ones to complete the conditioning. However, this technique requires continuous disconnections of the prosthetic components, which may lead to possible damage of peri-implant soft tissues Moreover, the number of appointments needed to obtain the right conditioning increases [23].

In view of the aforesaid, identifying the desired emergence profile of the customized healing abutment pre-operatively might improve the technique for a predictable result [24]. In 2016, Alshhrani et al. applied a new digital procedure to produce a personalized healing abutment [25]. The manufacturing process of this custom screw was performed by means of a scanner, the CAD/CAM method [26] and a virtual diagnostic wax-up. This technique significantly reduced chair time, giving the custom abutment the task of conditioning. Then, temporary crowns were used to verify the virtually designed emergence profile replicated in the definitive prosthesis.

In 2016, Joda et al. individualized the profile of the healing screws relying on the contralateral teeth acquired from cone beam computed tomography CBCT images [8]. The Digital Imagine and communication in medicine DICOM files of the CBCT scan were overlapped with the STL files of the intraoral digital impression after implant placement. The virtual segmentation of the digitally inverted tooth was transferred to CAD/CAM processing to obtain the customized healing abutment. No further conditioning was necessary and the obtained aesthetic result proved to be optimal. 

In the present study the shape of the standard healing screw was customized and positioned at the surgical stage, providing a transgingival healing. The results confirmed the data available in the literature [8,21,22,25].

The FIPS score was used to evaluate soft tissues, as it is a reproducible, objective and reliable evaluation instrument for assessing soft tissue around fixed implant restorations in premolar and molar sites. In 2018, Joda et al. assessed the reproducibility of the FIPS score and it showed strong statistical results, independent of the operator’s level of experience. In contrast to most implant indices, FIPS is defined by only five variables in order to limit bias. However, the FIPS score does not consider the implant connection, which can affect hard and soft tissue maturation. Furthermore, the original score does not consider hard and soft tissues volume, which may constitute an additional bias. To avoid such limitations, only patients presenting with no need of hard and soft tissue augmentation procedures were enrolled [27]. 

The test group showed no inflammation and a natural gingival architecture. FIPS values indicated higher scores when anatomically customized healing abutments were used (9.1 ± 0.9), compared to control cases (7.1 ± 0.9). These results confirmed the results of in vitro study, and therefore proved the higher aesthetic score in the test group. Placing a custom abutment at the surgical stage reduced contamination during prosthetic steps with biological advantages [28].

The NRS values for the assessment of perceived pain at time of crown insertion were significantly lower in the test group compared to the control group. It is noteworthy that local anesthesia was never used to reduce patient discomfort.

The shape of the tested healing screws was obtained from a digital library containing emergence profiles of standard dental elements, reducing the technical production time, and consequently, the costs for the clinician. Using the digital library allowed us to extend the protocol proposed by Joda and coworkers [8] in those cases where the contralateral element was not present. Furthermore, the present study did not require a CBCT scan to produce a virtual diagnostic model. The CBCT was requested only for second level investigations regarding the residual bone volume or for the identification of noble structures. Therefore, this system is easily applicable in clinical practice. 

Treatment time also proved to be widely reduced, thanks to the possibility of avoiding the provisional step. Indeed, the customized healing screws allow tissue conditioning, so that temporary restorations are no longer required. In the control cases the clinician had to administer local anesthetic due to the pain resulting from the compression of tissues (mean 5.5 ± 1.6 on the NRS scale) and frequently had to take an intraoral radiograph to verify the matching. 

The tissues were allowed to heal according to the final conformation without undergoing biological or mechanical trauma during the prosthetic phases. 

The data gained from this study represent only preliminary results obtained from a limited sample. In order to verify these results, a comparative analysis with a larger number of implants should be considered for further findings.

## 5. Conclusions

Within the limitations of the present study, it can be concluded that CAD/CAM customized healing abutments may require less steps for prosthetic finalization compared to standard healing abutments customized step by step with composite. The test group obtained higher FIPS scores than the control group. At the same time, pain perceived by the patients was lower in the test group during the early phases, however, it was not significantly different compared to the control group after 24 h.

## Figures and Tables

**Table 1:** Distribution of patients and teeth in test and control group.

Experimental Group	Patients ID	Gender	Age	Surgical Site
Test	1	F	63	46
Test	2	F	72	46
Test	3	M	45	37
Test	4	M	58	36
Test	5	F	75	35
Test	6	M	69	46
Test	7	F	68	16
Test	8	F	66	16
Test	9	M	54	14
Test	10	F	39	25
Control	11	F	44	26
Control	12	M	54	26
Control	13	F	56	46
Control	14	F	37	46
Control	15	F	58	36
Control	16	F	78	36
Control	17	M	59	35
Control	18	F	69	47
Control	19	M	79	15
Control	20	M	72	37

**Table 2:** Values of the functional implant prosthodontics score (FIPS) and pain in the control group.

CONTROL	FIPS	PAIN T0	PAIN T1	PAIN T2
1	6	8	5	4
2	7	6	3	0
3	7	5	4	0
4	6	7	4	0
5	8	3	0	0
6	8	4	2	0
7	8	4	1	0
8	6	7	4	0
9	8	6	3	0
10	7	5	1	0
AVERAGE	7.1	5.5	2.7	0.4
SD	0.9	1.6	1.6	1.3
MEDIAN	7	5.5	3	0

**Table 3:** Values of FIPS and pain in the test group.

TEST	FIPS	PAIN T0	PAIN T1	PAIN T2
1	9	0	0	0
2	10	0	0	0
3	8	1	0	0
4	10	0	0	0
5	8	2	0	0
6	10	0	0	0
7	8	0	0	0
8	9	1	0	0
9	9	1	0	0
10	10	0	0	0
AVERAGE	9.1	0.5	0	0
SD	0.9	0.7	0	0
MEDIAN	9	0	0	0
